# The Efficacy of *Tripterygium* Glycosides Combined with LMWH in Treatment of HSPN in Children

**DOI:** 10.1155/2021/7223613

**Published:** 2021-10-21

**Authors:** Yan Jin, Yanzheng Wang, Sai Wang, Qiongqiong Zhao, Donghua Zhang, Xuan Feng

**Affiliations:** ^1^Child Healthcare Department, Rizhao Hospital of TCM, Rizhao 276800, China; ^2^Department of Clinical Laboratory, Yantaishan Hospital, Yantai 264000, China; ^3^Department of Critical Medicine, Affiliated Qingdao Central Hospital, Qingdao University, Qingdao 266000, China; ^4^Department of Pediatrics, Zhangqiu District People's Hospital, Jinan 250200, China; ^5^Tumor-Chemotherapy Department, Zhangqiu District People's Hospital, Jinan 250200, China; ^6^Department of Outpatient, Qilu Hospital (Qingdao), Cheeloo College of Medicine, Shandong University, Qingdao 266035, China

## Abstract

**Objective:**

This study aimed to explore the clinical efficacy and relevant mechanism of *Tripterygium* glycosides combined with low molecular weight heparin calcium (LMWH) in the treatment of Henoch–Schönlein purpura nephritis (HSPN) in children.

**Methods:**

64 cases of children patients with HSPN treated at Qilu Hospital (Qingdao) from January 2015 to May 2020 were selected and randomly divided into the control group and the observation group and 32 cases in each group. Conventional medical treatment was applied in the two groups, besides which the control group was given LMWH while the observation group was given *Tripterygium* glycosides based on the control group. The clinical efficacy and the indexes of clinical symptoms of the two groups were compared. Immune globulin level, fibrinogen content (FIB), prothrombin time (PT), platelet level (PLT), and activated partial thromboplastin time (APTT) level of the two groups were compared before and after the treatment.

**Results:**

The total effective rate in the observation group was significantly higher than that of the control group, and the recurrence rate in the observation group was lower than that in the control group. After treatment, urine red blood cell count and 24 h urine protein were obviously better than those of the control group. There was no statistically significant difference in PT between the two groups of children before and after treatment. The levels of PLT and FIB in the two groups of patients after treatment were significantly lower than before treatment, and the PLT levels in the observation group were lower than those in the control group.

**Conclusion:**

The combination of *Tripterygium* glycosides and LMWH had good clinical effects in the treatment of children with HSPN, and it could improve the clinical symptoms, the mechanism of which might be related to the increase of PT, a decrease of PLT, and the improvement of coagulation function.

## 1. Introduction

Henoch–Schönlein purpura (HSP) in children is a systemic inflammatory vascular disease mediated by immunoglobulin A. The clinical manifestations of children with HSP include skin purpura, arthritis, hemorrhagic gastroenteritis, and renal damage, and in some cases, manifest as asymptomatic urine abnormalities [1]. Kidney damage is a common secondary injury in HSP, with a higher incidence of Henoch–Schönlein purpura nephritis (HSPN), often transient hematuria, accompanied by varying degrees of renal disease and functional impairment [2, 3]. HSPN tends to occur in children under 10 years of age, and it accounts for about 8% of pediatric urinary system patients [4]. The incidence rate in boys is higher than that in girls [5]. Most of the children show self-limiting characteristics and can be cured within a few weeks after the onset. However, about one-half of the children still have recurrent attacks [6]. Recent studies have shown that about 15% of children with HSPN develop persistent nephropathy, and 8% of children with HSPN develop renal failure. Therefore, the treatment of children with HSPN should be taken seriously to get the desired outcomes [7]. At present, glucocorticoids, immunosuppressants, and other drugs are often used to treat pediatric HSPN, but these treatments have obvious defects such as prolonged treatment course, severe adverse reactions, and recurrence [8].

*Tripterygium wilfordii* is a vine-like plant that grows in southeastern of China and has been used in traditional Chinese medicine for thousands of years. *Tripterygium* glycosides are natural active ingredient extracted from *Tripterygium wilfordii* [9]. It has a variety of pharmacological functions, such as detoxification, invigorating blood, preventing inflammation, and antifertility [10]. *Tripterygium* glycosides have been used in the treatment of HSPN in China. A previous study has suggested that *Tripterygium* glycosides can relieve hematuria and proteinuria in immunoglobulin A deposition nephropathy and diabetic nephropathy [11]. In addition, *Tripterygium* glycosides can enhance the effect of thiamazole and prednisone in the treatment of hyperthyroidism [12]. Moreover, *Tripterygium* glycosides have been used to reduce proteinuria and protect the kidney for more than 20 years [13, 14].

In this study, *Tripterygium* glycosides and low molecular weight heparin calcium (LMWH) were used to treat HSPN to evaluate its clinical efficacy on HSPN. At the same time, the changes in the levels of immunoglobulin and thrombin in patients were compared, and the related mechanisms of clinical efficacy were explored, which aims to provide a scientific basis for clinically formulating reasonable and effective treatment plans.

## 2. Materials and Methods

### 2.1. Clinical Information

Sixty-four children with HSPN admitted at Qilu Hospital (Qingdao), Cheeloo College of Medicine, Shandong University, Qingdao, Shandong, China, from January 2015 to May 2020, were selected and divided into the control group and observation group, with 32 cases in each group. In the control group, there were 20 males and 12 females; they were 4–15 years old, with an average of (9.1 ± 2.8) years old; the course of the disease was 5–30 days, with an average of (17.5 ± 8.0) d. In the observation group, there were 22 males and 10 females; the age was 3–13 years, with an average of (7.9 ± 2.7) years; the course of the disease was 7–25 days, with an average of (14.4 ± 5.9) d. There is no statistical difference of children in the above clinical data (*P* > 0.05).

### 2.2. Inclusion Criteria and Exclusion Criteria

  Inclusion criteria: (I) children aged 2–15 years; (II) no immunosuppressive agents, glucocorticoids, and nonsteroidal drugs were used 2 weeks before treatment, such as *Tripterygium* glycoside, cyclophosphamide, or mycophenolate mofetil; (III) the course of the disease was less than 2 months; (IV) kidney pathological grade is grade I-II; (V) all guardians of the children signed the informed consent form; (VI) patients had no surgical operation history caused by HSPN.
  Exclusion criteria: (I) severe heart and cerebrovascular diseases and insufficient liver and kidney function; (II) children with D-dimer or fibrinase less than normal; (III) children with allergies to the drugs used in this study; (IV) switch to or add with another immunosuppressive agent (mycophenolate mofetil, cyclosporine A, and tacrolimus) during treatment; (V) diagnosed with hypercalciuria; (VI) diagnosed with other systemic diseases that may affect renal function (systemic lupus erythematosus).

The study got the approval of the Ethics Committee of Qilu Hospital (Qingdao), Cheeloo College of Medicine, Shandong University, Qingdao, China. Patients' family members fully knew the study process and they signed informed consent forms.

### 2.3. Interventions

Both groups of patients were given conventional medical treatments such as antihistamine therapy, hormone therapy, antiplatelet aggregation therapy, and supportive medical therapy, and dipyridamole tablets were taken orally (Shanghai Xinyi Jiufu Pharmaceutical Co., Ltd., National Medicine Standard H44020689, 25 mg/tablet), 1 mg/kg each time, 3 times a day.

On this basis, children in the control group were given low molecular weight heparin calcium (Shenzhen Saibaoer Biopharmaceutical Co., Ltd., 0.5 mL: 5000 AXaU) 100 IU/kg subcutaneously, once a day, for continuous use of 12 weeks. Children in the observation group were given *Tripterygium* glycosides tablets (Zhejiang Prokangyu Natural Medicine Co., Ltd., National Medicine Standard Z33020778, 10 mg) on the basis of control group, 1.5 mg/kg each time, and it was given 30 minutes after breakfast, lunch, and dinner, respectively. The treatment course of both groups was 12 weeks.

### 2.4. Clinical Treatment Effect

The clinical efficacy evaluation criteria are as follows [15]: (I) if clinical symptoms and signs, biochemical tests, blood pressure, and urine protein return to normal, hematuria disappears, and no recurrence within 3 months, it was considered to be cured; (II) if clinical symptoms and signs, biochemical tests, blood pressure, and urine protein have basically returned to normal, hematuria disappears, and a small number of recurrences within 3 months, it was considered to be excellent; (III) if clinical symptoms and signs, biochemical tests, blood pressure, and urine protein are all normal, and as a result, hematuria has disappeared, it was considered to be effective; (IV) if clinical symptoms and signs, biochemical tests, blood pressure, urine protein, and hematuria have not improved and the condition even worsened, it was considered to be invalid. Total effective rate = (cure + excellent + effective) number of cases/total number of cases × 100%.

### 2.5. Renal Function Index and Coagulation Functional Index

The fasting elbow venous blood and morning urine were collected before and after the treatment of the child, and a fully automatic biochemical analyzer (American Beckman AU-5800) was used to detect renal function indicators (24-hour urine protein). Urine microscopic was used to count the red blood cell (urine RBC). The automatic coagulation analyzer (model: ACL7000; Beckman Coulter Co., Ltd., USA) and supporting kits were used to detect coagulation function indexes: fibrinogen content (FIB), prothrombin time (PT), platelet level (PLT), and activated partial thromboplastin time (APTT). Record the occurrence of adverse reactions during the hospitalization. The treatment course of the two groups was 12 weeks, and the follow-up was one year.

### 2.6. Judgment Criteria for Kidney Damage

After 4 weeks of follow-up treatment and 3 months, urine was tested to determine the kidney damage. The normal value of urinary transferrin is 0.001–5 mg/L. The normal value of urinary N-acetyl-*β*-D-glucosaminidase is 0.3–12 IU/L. The normal value of urinary *β*2-microglobulin is 0.1–25 mg/L. If any of the indicators is abnormal, it is considered that there is kidney damage.

### 2.7. Statistical Methods

SPSS 19.0 was used for data analysis, data were expressed as mean ± standard deviation (*χ* ± *S*), comparison between two groups was by the *t*-test, and comparison between groups was by thee *χ*^2^ test. *P* < 0.05 indicates that the difference is statistically significant.

## 3. Results

### 3.1. Comparison of Baseline Data

As given in Table 1, after treatment in the observation group, there were 15 children with HSPN cured patients, 8 excellent patients, 7 effective patients, and 2 invalid patients; the total effective rate was 94%. In the control group after treatment, there were 10 cases of HSPN cured patients, 8 cases of excellent patients, 4 cases of effective patients, and 10 cases of invalid patients; the total effective rate was 69%. The total effective rate in the observation group was significantly higher than that in the control group (*P* < 0.05). After the treatment, all children were followed up for one year, and it was found that 15 cases in the control group relapsed, while 4 cases in the observation group relapsed. The recurrence rate in the observation group was significantly lower than that in the control group (*P* < 0.05).

### 3.2. Comparison of Urine Red Blood Cell Count and 24-Hour Urine Protein

It can be seen from Table 2 that, before treatment, there was no statistically significant difference in urine red blood cell count and 24-hour urine protein between the two groups (*P* > 0.05). After treatment, the above indicators of the two groups were significantly reduced (*P* < 0.05), and the two indexes of the observation group were significantly lower than those of the control group (all *P* < 0.05).

### 3.3. Comparison of Coagulation Function Index

There was no statistically significant difference in PT between the two groups of children before and after treatment (*P* > 0.05). After treatment, the APPT levels of the control group did not change significantly from before treatment (*P* > 0.05), while the APPT levels of the observation group were significantly increased (*P* < 0.05), and the difference between the two groups was significant (*P* < 0.05, Table 3). The levels of PLT and FIB in two groups of patients after treatment were lower than before treatment (*P* < 0.05), and the PLT levels in the observation group were lower than those in the control group (*P* < 0.05). There was no significant difference in the FIB level between the two groups after treatment (*P* > 0.05) (Table 4).

### 3.4. Comparison of Adverse Reactions and Kidney Function Damage

During the treatment period, 2 cases of adverse reactions occurred in the observation group (1 case of nausea and 1 case of diarrhea and were resolved spontaneously); the incidence rate in the observation group was 6.25%. 3 cases of adverse reactions occurred in the control group (2 cases of nausea and 1 case of dizziness, all of them spontaneously); the incidence rate of adverse reactions in the control group was 9.37%. There was no significant difference in the incidence of kidney damage between the two groups of children before treatment and after 4 weeks of treatment (*P* > 0.05). Two cases in the observation group were found lost to follow-up after 3 months of treatment. The incidence of kidney damage in the control group was significantly higher than that in the observation group (*P* < 0.05, Table 5).

## 4. Discussion

According to a survey on the spectrum of childhood glomerular diseases performed in China from 2004 to 2014, HSPN (13%) and lupus nephritis (9%) are the most common secondary glomerular diseases in children. Purpuric nephritis (23%) is the most common pathological pattern observed in young children (0–12 years old) [16]. The clinical features of HSPN are hematuria and urine protein, some of which are accompanied by hypertension and renal insufficiency, which seriously affect children's physical and mental health. The increasing incidence of HSPN has attracted great attention in clinical treatment, and previous studies reported that the immature immune system of infants and young children will trigger HSPN, and the younger the child, the greater the probability of HSPN [17]. Long-term persistent proteinuria and hematuria can cause renal failure and cause irreversible damage to patients. Therefore, early diagnosis and early treatment are essential for children with HSPN.

HSPN is a systemic inflammatory response of small blood vessels, and its pathogenesis is not yet clear. There is direct evidence that food, environmental factors, infections, chemical exposure, drug allergies, insect bites, and other factors are all related to HSPN [18]. Some scholars believe that the above factors may cause the activated complement and immune complexes to be deposited in the glomerular mesangium and ultimately affect the normal physiological and metabolic mechanisms of blood coagulation to trigger the disease [19]. The most manifested is the hypercoagulable state of the blood and the disorder of the coagulation and fibrinolysis system. In addition, platelet activation and aggregation complement activation and abnormal humoral immune mechanisms are closely related to the occurrence and development of the disease [20, 21]. Disorders of the coagulation and fibrinolysis system cause abnormal elevation of PT and APTT and a significant decrease in D-D and FIB, which are risk factors for abnormal coagulation and hemostasis, platelet aggregation, inflammatory effects, and glomerular diseases [22]. Therefore, the primary goal of clinical treatment should be to improve coagulation function.

According to traditional Chinese medicine (TCM) dialectics, HSPN belongs to the categories of “purpura” and “hematuria” [23, 24]. Its pathogenesis is mainly due to blockage of the collaterals and blood-heat delusion, which is a symptom of deficiency and excess. Children are the body of immature Yin and Yang, with innate endowment and insufficient Qi machine, the onset of the disease is rapid, and the disease is difficult to heal [25]. *Tripterygium wilfordii* has the effects of bitter, cold, cool in nature, with great toxicity, and returns to the liver and kidney meridians. It has the effects of dispelling wind and dampness, promoting blood circulation, dredging collaterals, reducing swelling and pain, killing insects, and detoxifying [26]. *Tripterygium* glycosides are a glycoside extracted from the roots of *Tripterygium*, and its main component is epoxy diterpene lactones. Previous reports showed that *Tripterygium* glycosides had potent anti-inflammatory and immunosuppressive effects [27]. *Tripterygium* glycosides tablets have been indicated to reduce the inflammatory response in rat lung tissue [28]; it can upregulate IL-10 expression and TNF-*α* levels in the rat arthritis model [29].

This study shows that the observation group has better efficacy than the control group, and the recurrence rate is significantly lower than that of the control group. It shows that *Tripterygium* glycosides combined with LMWH are more effective than LMWH in treating children with HSPN. After treatment, the improvement of clinical symptoms of the observation group was better than that of the control group, indicating that *Tripterygium* glycosides combined with LMWH had a better effect on improving the clinical symptoms of HSPN. The total effective rate of *Tripterygium* glycosides combined with LMWH treatment was significantly higher than that of LMWH treatment alone, indicating that the combination of Chinese and Western medicines is more effective than treatment with Western medicines. After treatment in the two groups, the urine red blood cell and 24-hour urine protein content were significantly reduced, indicating that *Tripterygium* glycosides combined with LMWH can further improve microcirculation and hemorheology, effectively alleviate the symptoms of proteinuria and hematuria, and improve renal function. PLT and FIB were significantly reduced, APTT was significantly increased, and the improvement of various indicators in the observation group was significantly better than that of the control group after treatment, indicating that the combination of *Tripterygium* glycosides combined with LMWH has a stronger effect of promoting blood circulation and removing blood stasis than LMWH. LMWH and *Tripterygium* glycosides have a synergistic effect, which significantly improves the patient's hypercoagulable state, hemorheology, and microcirculation, and prevents the exacerbation of the disease. The possible reasons are as follows: (1) *Tripterygium* glycosides significantly improve glomerular capillary permeability, can effectively reduce the level of urinary protein, and effectively reverse its pathological changes; (2) LMWH can produce various coagulation factors. At the same time, inhibition has a significant improvement effect on the permeability of the filter membrane and improves kidney function. However, there is still room for improvement in this study. To begin with, the insufficient sample size may lead to a large probability of error in data deviation, so we hope to increase the sample size in future research to reduce the deviation of results. Moreover, the reasons for the decrease of adverse drug reactions shall be further explored to stabilize the efficacy of drugs better. These are the directions of our follow-up and improvement, so as to find a better treatment for this disease.

The results of this study showed that the total effective rate of treatment in the observation group was significantly higher than that in the control group, and the recurrence rate of the observation group was lower than that in the control group. After treatment, the urine red blood cell count and 24-hour urine protein were significantly lower than that in the control group. The levels of PLT and FIB in two groups of patients after treatment were lower than before treatment; the PLT levels in the observation group were lower than those in the control group. There was no significant difference in the incidence of kidney damage between the two groups of children before treatment and after 4 weeks of treatment. The incidence of kidney damage in the control group was significantly higher than that in the observation group. It shows that the combination of *Tripterygium* glycoside tablets and LMWH in the treatment of children with HSPN has significant efficacy and high safety and has the potential for clinical application.

## Figures and Tables

**Table 1:** Comparison of clinical efficacy between two groups.

Group	Cases (*n*)	Cure	Excellent	Effective	Invalid	Total effective rate (%)	Recurrence rate
Control group	32	10 (31%)	8 (25%)	4 (12.5%)	10 (31%)	69	15 (47%)
Observation group	32	15 (47%)	8 (25%)	7 (22%)	2 (6%)	94	4 (12.5%)

**Table 2:** Comparison of the indexes of urine RBC and 24 h urine protein between two groups.

Group	Cases (*n*)	Urine RBC (*n*/*μ*l)		24 h urine protein (g)	
		Before treatment	After treatment	Before treatment	After treatment
Control group	32	219.9 ± 33.01	94.83 ± 8.28^a^	0.17 ± 0.02	0.13 ± 0.01^a^
Observation group	32	205.0 ± 20.28	43.40 ± 9.22^a^	0.17 ± 0.01	0.07 ± 0.02^a^
*T*		0.6661	7.191	0.7276	5.376
*P*		0.5418	0.002	0.5072	0.0058

^a^Compared with before treatment, *P* < 0.05.

**Table 3:** Comparison of the indexes of PT and APTT between two groups.

Group	Cases (*n*)	PT (s)		APTT (s)	
		Before treatment	After treatment	Before treatment	After treatment
Control group	32	12.4 ± 1.01	12.7 ± 0.96	23.73 ± 0.80	25.07 ± 0.48
Observation group	32	12.63 ± 0.74	13.87 ± 0.35	23.73 ± 0.97	28.27 ± 0.57^a^
*T*		0.3222	1.969	0	7.496
*P*		0.7634	0.1203	>0.99	0.0017

^a^Compared with before treatment, *P* < 0.05.

**Table 4:** Comparison of the indexes of PLT and FIB between two groups.

Group	Cases (*n*)	PLT (х 10^10^/L)		FIB (g/L)	
		Before treatment	After treatment	Before treatment	After treatment
Control group	32	28.67 ± 0.99	26.39 ± 0.48^a^	3.38 ± 0.10	2.78 ± 0.07^a^
Observation group	32	28.68 ± 1.22	24.15 ± 0.44^a^	3.4 ± 0.11	2.70 ± 0.06^a^
*T*		0.011	5.959	0.2437	1.488
*P*		0.9917	0.004	0.8194	0.2109

^a^Compared with before treatment, *P* < 0.05.

**Table 5:** Comparison of kidney damage between the two groups.

Group	Cases (*n*)	Before treatment		After 4 weeks of treatment		After 3 months of treatment	
		Kidney damage	No kidney damage	Kidney damage	No kidney damage	Kidney damage	No kidney damage
Control group	32	3 (9.37%)	29 (90.62%)	8 (25%)	24 (75%)	13 (40.62%)	19 (59.38%)
Observation group	32	2 (6.25%)	30 (93.75%)	6 (18.75%)	26 (81.25%)	3 (10%)^a^	27 (90%)

^a^Compared with the control group, *P* < 0.05.

## Data Availability

The datasets used to support the findings of this study are available from the corresponding author upon request.

## Supplementary Materials

Supplementary Materials The flowchart of *Tripterygium* glycosides combined with LMWH in treatment of HSPN in children.

## References

[1] Yu Y., Chen J., Yin H. (2020). Efficacy of steroid and immunosuppressant combined therapy in Chinese patients with Henoch-Schönlein purpura nephritis: a retrospective study. *International Immunopharmacology*.

[2] Delbet J. D., Hogan J., Aoun B. (2017). Clinical outcomes in children with Henoch-Schönlein purpura nephritis without crescents. *Pediatric Nephrology*.

[3] Jelusic M., Sestan M., Cimaz R., Ozen S. (2019). Different histological classifications for Henoch-Schönlein purpura nephritis: which one should be used?. *Pediatric Rheumatology*.

[4] Leung A. K., Wong A. H., Barg S. S. (2017). Proteinuria in children: evaluation and differential diagnosis. *American Family Physician*.

[5] Cheng S., Zhu C.-H., Zhang A.-H., Huang S.-M. (2020). MiR-29b expression is altered in crescent formation of HSPN and accelerates Ang II-induced mesangial cell activation. *World Journal of Pediatrics*.

[6] Shah R., Ramakrishnan M., Vollmar A., Harrell A., Van Trump R., Masoud A. (2017). Henoch-schonlein purpura presenting as severe gastrointestinal and renal involvement with mixed outcomes in an adult patient. *Cureus*.

[7] Ding Y. Z. W. S., Ren X. Q., Liu Y. Q. (2012). Curative effect observation of Tripterygium wilfordii combined heat stop bleeding side, xiangdan injection in treatment of children with purpura nephritis. *Zhongguo Zhongxiyijiehe Zazhi (Chinese Journal of Integrated Traditional and Western Medicine)*.

[8] Subspecialty Group of Renal Diseases tSoPCMA (2009). [Evidence-based guideline for diagnosis and treatment of Henoch-Schonlein purpura nephritis]. *Zhonghua Er Ke Za Zhi*.

[9] Xu X., Li Q. J., Xia S., Wang M. M., Ji W. (2016). Tripterygium glycosides for treating late-onset rheumatoid arthritis: a systematic review and meta-analysis. *Alternative Therapies in Health and Medicine*.

[10] Fang T., Liu L., Liu W. (2020). Network pharmacology-based strategy for predicting therapy targets of Tripterygium wilfordii on acute myeloid leukemia. *Medicine*.

[11] Liu X., Gao C., Liu X., Gao T. (2019). Efficacy and safety of tripterygium glycosides for graves ophthalmopathy. *Medicine*.

[12] Xie C., He C., Gao J., Jia S. (2020). Efficacy and safety of tripterygium glycosides in the treatment of hyperthyroidism: a systemic review and meta-analysis. *Medicine*.

[13] Lu Z.-Y., Yang H.-F., Peng Y. (2016). Treatment of fibrillary glomerulonephritis by corticosteroids and tripterygium glycoside tablets: a case report. *Chinese Journal of Integrative Medicine*.

[14] Jin D., Yu M., Li X., Wang X. (2021). Efficacy of Tripterygium wilfordii hook F on animal model of diabetic kidney diseases: a systematic review and meta-analysis. *Journal of Ethnopharmacology*.

[15] Hennies I., Gimpel C., Gimpel C. (2018). Presentation of pediatric Henoch-Schönlein purpura nephritis changes with age and renal histology depends on biopsy timing. *Pediatric Nephrology*.

[16] Nie S., He W., Huang T. (2018). The spectrum of biopsy-proven glomerular diseases among children in China: a national, cross-sectional survey. *Clinical Journal of the American Society of Nephrology*.

[17] Tan J., Tang Y., Xu Y. (2019). The clinicopathological characteristics of henoch-schönlein purpura nephritis with presentation of nephrotic syndrome. *Kidney and Blood Pressure Research*.

[18] Sotoodian B., Robert J., Mahmood M. N., Yacyshyn E. (2015). IgA cutaneous purpura post-renal transplantation in a patient with long-standing IgA nephropathy: case report and literature review. *Journal of Cutaneous Medicine and Surgery*.

[19] Couch C. E., Schubert M. S. (2014). Functional antipolysaccharide immunoglobulin deficiency, recurrent pneumococcal sepsis, and hypergammaglobulinemic purpura. *The Journal of Allergy and Clinical Immunology: In Practice*.

[20] Yang X.-Q., Huang Y.-J., Zhai W.-S. (2019). Correlation between endocapillary proliferative and nephrotic-range proteinuria in children with Henoch-Schönlein purpura nephritis. *Pediatric Nephrology*.

[21] Chen X. Y., Yi Z. W., He Q. N. (2008). [Relationship between leukotrienes and clinical, pathological features of Henoch-Schoenlein purpura nephritis in children]. *Zhonghua Er Ke Za Zhi*.

[22] Agrawal S., Jain A., Rajput A. (2014). Leukocytoclastic vasculitis and acute allergic interstitial nephritis following ceftriaxone exposure. *Journal of Pharmacology and Pharmacotherapeutics*.

[23] Zhang J., Lv J., Pang S. (2018). Chinese herbal medicine for the treatment of Henoch-Schonlein purpura nephritis in children: a prospective cohort study protocol. *Medicine*.

[24] Ding D., Yan H., Zhen X. (2014). Effects of Chinese herbs in children with Henoch-Schonlein purpura nephritis: a randomized controlled trial. *Journal of Traditional Chinese Medicine*.

[25] Jin Z. D., Wang S. C., Sun Y. Q. (2003). [Effect of danshao granule on serum superoxide dismutase activity and malonyldialdehyde content in children with Henoch-Schonlein purpura nephritis]. *Zhongguo Zhong Xi Yi Jie He Za Zhi*.

[26] Lin N., Zhang Y. Q., Jiang Q. (2020). Clinical practice guideline for tripterygium glycosides/tripterygium wilfordii tablets in the treatment of rheumatoid arthritis. *Frontiers in Pharmacology*.

[27] Cai A., Qi S., Su Z., Shen H., Ma W., Dai Y. (2015). Tripterygium glycosides inhibit inflammatory mediators in the rat synovial RSC-364 cell line stimulated with interleukin-1*β*. *Biomedical Reports*.

[28] Wan L., Liu J., Huang C.-B. (2013). Effect of tripterygium glycosides on pulmonary function in adjuvant arthritis rats. *Journal of the Chinese Medical Association*.

[29] Lei W., Jian L. (2012). Changes of CD4 (+) CD25 (+) regulatory T cells, FoxP3 in adjuvant arthritis rats with damage of pulmonary function and effects of tripterygium glycosides tablet. *The Internet Journal of Rheumatology*.

