# Supplementary figures and images for: The Efficacy of Tripterygium Glycosides Combined with LMWH in Treatment of HSPN in Children
Source: Evid Based Complement Alternat Med. 2021 Oct 21;2021:7223613. doi: 10.1155/2021/7223613 (PMC8553442; doi:10.1155/2021/7223613)

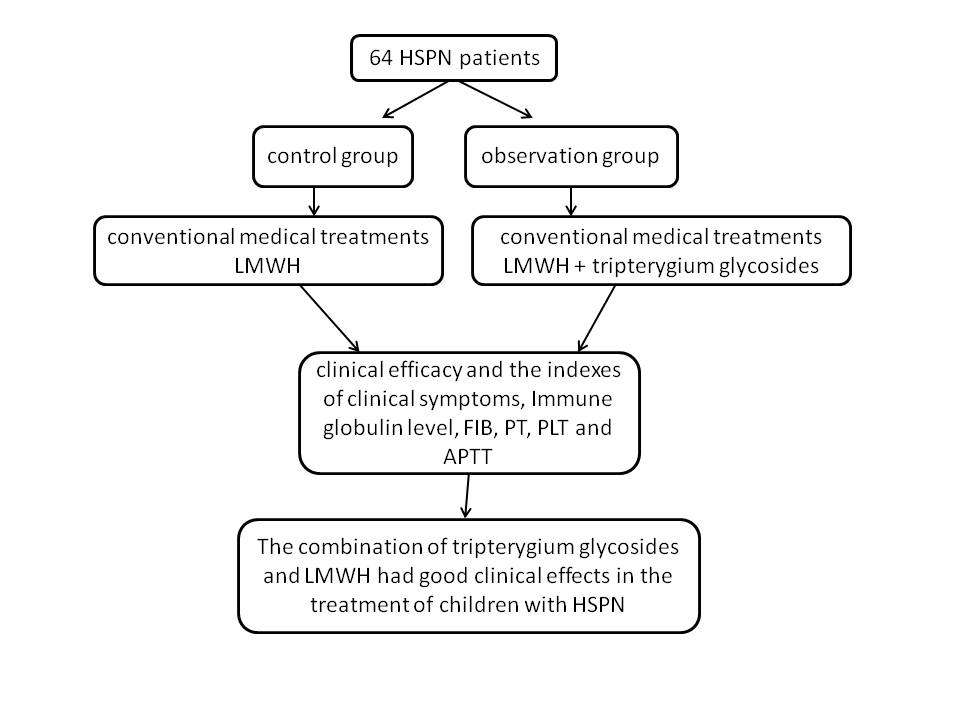

Supplement: Supplementary Materials — The flowchart of Tripterygium glycosides combined with LMWH in treatment of HSPN in children. [file 7223613.f1.jpg]
